# Prevalence, clinical characteristics, and independent predictors of uveitic macular edema in an Asian population: a retrospective cohort study

**DOI:** 10.1186/s12886-024-03447-0

**Published:** 2024-04-22

**Authors:** Usanee Tungsattayathitthan, Sukanda Jenjanya, Pitipol Choopong, Wilawan Sanphan, Nattaporn Tesavibul, Sutasinee Boonsopon

**Affiliations:** Department of Ophthalmology, Faculty of Medicine Siriraj Hospital, Mahidol University, 2 Wanglang Road, 10700 Bangkoknoi, Bangkok, Thailand

**Keywords:** Intermediate uveitis, Panuveitis, Posterior uveitis, Predictive factors, Prevalence, UME, Uveitic macular edema

## Abstract

**Background:**

To determine the prevalence, clinical characteristics, and independent predictors of uveitic macular edema (UME) in patients with intermediate, posterior and panuveitis.

**Methods:**

We retrospectively reviewed the records of patients with intermediate, posterior, and panuveitis who underwent macular assessment using optical coherence tomography between January 2015 and February 2020. The prevalence of UME and clinical characteristics of the patients were described. Predictors of UME were identified using multivariate regression analysis.

**Results:**

A total of 349 patients were included. The mean age was 41 years, female: male ratio was 1.3:1. The prevalence of UME was 51.9%. UME was found in 33.9%, 56.9%, and 54.1% of the intermediate, posterior, and panuveitis cases, respectively. Among patients with UME, 47% had infectious uveitis, 32.6% had idiopathic uveitis, and 20.4% had immune-mediated uveitis. Diffuse macular edema was the most frequently observed pattern (36.5%). Multivariate analysis showed that factors independently associated with UME included age at uveitis onset (adjusted odds ratio [aOR] 1.01, 95% confidence interval [CI] 1.00-1.03, *P* = 0.036), PU and panuveitis compared with intermediate uveitis (aOR 2.09, 95% CI 1.14–3.86, *P* = 0.018), and infectious uveitis compared with noninfectious uveitis (aOR 2.13, 95% CI 1.34–3.37, *P* = 0.001).

**Conclusions:**

Increasing age at uveitis onset, posterior/panuveitis, and infectious etiology are predictive factors for UME in patients with intermediate, posterior and panuveitis.

## Background

Uveitic macular edema (UME) is a frequent and potentially severe complication of uveitis that can lead to visual impairment and eventually blindness [1, 2]. Among uveitis patients with UME, the prevalence of visual impairment has been reported to range from 9 to 66%, with a higher percentage in those with intermediate, posterior, and panuveitis [1, 3–6]. UME is associated with intraocular inflammation, which can occur at any location within the eye, and its etiology can be related to infection, immune processes, masquerade, or idiopathic. UME can persist even after uveitis has subsided and its pathogenesis is not entirely understood. However, the suspected mechanism is the breakdown of the blood-retina barrier, leading to increased inflammatory mediators, increased vascular permeability, and extravasation of fluid, resulting in fluid accumulation in the macular region [7].

Although several studies have identified factors associated with visual loss or complications in patients with uveitis, those specifically associated with UME development have not been well characterized in the Asian population. Whether any of the presenting characteristics can predict the occurrence of UME remains unclear. To address this question, we conducted a study to determine the predictive factors of developing UME among patients with intermediate, posterior, and panuveitis.

## Methods

This retrospective study was conducted at the uveitis clinic of Siriraj Hospital, a tertiary referral center in Thailand. Patients who presented with intermediate uveitis (IU), posterior uveitis (PU), or panuveitis between January 2015 and February 2020 were enrolled in this study. We excluded patients who had inadequate media clarity assessment and those who did not undergo foveal assessment using spectral domain optical coherence tomography (SD-OCT). Patients with coexisting conditions that can cause macular edema, including diabetes mellitus, retinal vascular occlusion, and wet age-related macular degeneration, were also excluded. If bilateral uveitis was present, only the characteristics of the right eye were analyzed. We recorded patients who had UME at presentation or developed UME during the follow-up period. For patients who developed recurrent UME, we only included the details of their first presentation with UME.

Demographic and clinical data, specifically age, sex, laterality, were collected. The etiology of uveitis was recorded and grouped into infectious and noninfectious uveitis, which included immune-mediated and idiopathic uveitis. The clinical course (acute, chronic, recurrence) and location of inflammation (IU, PU, panuveitis) were categorized according to The Standardization of Uveitis Nomenclature criteria [8]. Macular edema was defined as a central subfield thickness greater than 300 μm, assessed using SD-OCT (Spectralis; Heidelberg Engineering, Heidelberg, Germany) [9]. UME is classified into three subtypes: cystoid macular edema (CME), diffuse macular edema (DiffME), and serous retinal detachment (SRD) [4, 10, 11]. CME is defined as low-reflectivity intraretinal spaces that are clearly defined and separated by thin, highly reflective retinal tissues. DiffME is defined as an increased macular thickness and small, low-reflective areas with a spongy appearance in the retinal layers. SRD consists of a clear separation of the neuroretinal layer from the retinal pigment epithelium. The angle formed by these two layers is 20–30°, and a hyperreflectivity line clearly demonstrates the posterior border of the detached retina with attachment to the retinal pigment epithelium in the peripheral margin of the subretinal space [10, 12].

The demographic and clinical characteristics were compared between the UME and non-UME groups. The study protocol was approved by the Siriraj Institutional Review Board (SIRB) (approval no. SI 164/2020) following the tenets of the Declaration of Helsinki. The requirement for informed consent was waived by the SIRB.

### Statistical analysis

Descriptive statistics were used to summarize patient characteristics. Categorical data were compared using either the chi-square test or Fisher’s exact test, and the results are presented as numbers and percentages. Normally distributed continuous data were compared using the Student’s t test, and the results are reported as the mean ± standard deviation. Variables with a p value < 0.1 in univariate analysis were entered into a stepwise backward multivariate model to identify factors independently associated with UME. All statistical analyses were performed using IBM SPSS Statistics version 29 (IBM Corp., Armonk, N.Y., USA). A *P* value of less than 0.05 was considered statistically significant.

## Results

Three hundred forty-nine patients met the inclusion criteria and were enrolled in this study. A total of 181 patients (51.9%) had UME; of these, 70.2% had unilateral UME. The mean follow-up period for all patients was 40 ± 33.7 months. Table 1 summarizes the demographic data and baseline characteristics of all the patients with and without UME. Overall, the mean age (± SD) was 40.6 ± 16.6 years and 57.3% were female. A trend toward a higher mean age was observed in the UME group than in the non-UME group (42.2 ± 16.3 vs. 38.9 ± 16.7 years, *P* = 0.063).

**Table 1:** Demographic and baseline characteristics of all patients

Parameters	Total (*N* = 349)	UME (*n* = 181)	Non-UME (*n* = 168)	P value
Follow-up time (months), mean ± SD	39.95 ± 33.65	36.65 ± 30.60	43.49 ± 36.41	0.058
Age (years), mean ± SD	40.6 ± 16.6	42.2 ± 16.3	38.9 ± 16.7	0.063
Female gender, n (%)	200 (57.3)	97 (53.6)	103 (61.3)	0.150
Bilateral uveitis, n (%)	190 (54.4)	92 (50.8)	98 (58.3)	0.160
Chronic uveitis, n (%)	255 (73.1)	126 (69.6)	129 (76.8)	0.130
Location of inflammation, n (%)				
Intermediate uveitis	59 (16.9)	20 (11.1)	39 (23.2)	0.009*
Posterior uveitis	144 (41.3)	82 (45.3)	62 (36.9)	
Panuveitis	146 (41.8)	79 (43.7)	67 (39.9)	
*Type of inflammation, n (%)*				
Granulomatous inflammation	175 (50.1)	90 (49.7)	85 (50.6)	0.870
Nongranulomatous inflammation	174 (49.9)	91 (50.3)	83 (49.4)	
Etiology, n (%)				
Infection	131 (37.5)	85 (47.0)	46 (27.4)	0.001*
Immune-mediated	88 (25.2)	37 (20.4)	51 (30.4)	
Idiopathic	130 (37.3)	59 (32.6)	71 (42.3)	
Diagnosis, n (%)				
Tuberculosis	44 (12.6)	25 (13.8)	19 (11.3)	
ARN/PORN/CMVR	42 (12)	28 (15.5)	14 (8.3)	
Vogt-Koyanagi-Harada disease	27 (7.7)	16 (8.8)	11 (6.5)	
White dot syndrome	19 (5.4)	2 (1.1)	17 (10.1)	
Parasite-related uveitis	18 (5.2)	11 (6.1)	7 (4.2)	
Sarcoidosis	17 (4.9)	8 (4.4)	9 (5.4)	
Behçet’s disease	13(3.7)	6 (3.3)	7 (4.2)	
Syphilis	9 (2.6)	7 (3.9)	2 (1.2)	
HLA-B27/SNSA ^a^	7 (2)	1 (0.5)	6 (3.6)	
Toxoplasmosis	7 (2)	6 (3.3)	1 (0.6)	
Cat-scratch neuroretinitis	6 (1.7)	4 (2.2)	2 (1.2)	
Endogenous endophthalmitis	5 (1.4)	4 (2.2)	1 (0.6)	
Juvenile idiopathic arthritis ^b^	3 (0.9)	3 (1.7)	0	
Systemic lupus erythematosus	2 (0.6)	1 (0.5)	1 (0.6)	
Maximal macular thickness (µm), mean ± SD	358.11 ± 136.26	443.66 ± 142.29	265.94 ± 19.68	< 0.001*

ARN acute retinal necrosis; CMVR cytomegalovirus retinitis; HLA human leukocyte antigen; PORN progressive outer retinal necrosis; SD standard deviation; SNSA seronegative spondyloarthropathy; UME uveitic macular edema

^a^ Among 7 patients with HLA-B27/SNSA, 4 had intermediate uveitis, and 3 had panuveitis

^b^ Among 3 patients with JIA, 2 had intermediate uveitis, and 1 had panuveitis

The majority of patients presented with bilaterality (54.4%), a chronic course (73.1%), and panuveitis (41.8%). Granulomatous inflammation was observed in 50.1% of patients. IU was observed in 16.9%, PU in 41.3%, and panuveitis in 41.8% of all patients, with a higher proportion of patients with PU and panuveitis in the UME group than in the non-UME group. Statistically significant differences were found between the two groups with respect to the location of the inflammation (*P* = 0.009). When evaluating the location of uveitis in relation to IU, PU, and panuveitis, UME occurred in 33.9%, 56.9%, and 54.1% of patients with IU, PU, and panuveitis, respectively.

The etiology of uveitis was established in 219 patients (62.8%). One hundred thirty-one (37.5%) were diagnosed with an infectious etiology and 88 (25.2%) were diagnosed with a recognized immune-mediated process. The remaining 130 patients (37.3%) were classified as idiopathic. Among the 181 patients with UME, 85 (47.0%) had infectious uveitis, 37 (20.4%) had a recognized immune-mediated process, and 59 (32.6%) had idiopathic inflammation. Regarding the etiology of uveitis, UME was observed in 64.9% (85 out of 131) and 44% (96 out of 218) of the patients with infectious and noninfectious uveitis, respectively. Based on the diagnosis, UME occurred in more than 50% of patients with juvenile idiopathic arthritis (3 of 3 [100%]), toxoplasmosis retinochoroiditis (6 of 7 [85.7%]), endogenous endophthalmitis (4 of 5 [80%]), syphilitic uveitis (7 of 9 [77.8%]), viral posterior/panuveitis (28 of 42 [66.7%]), cat-scratch neuroretinitis (4 of 6 [66.7%]), parasite-related uveitis (11 of 18 [61.1]), Vogt-Koyanagi-Harada disease (16 of 27 [59.3%]), and tuberculous uveitis (25 of 44 [56.8%]).

The mean maximum macular thickness was significantly greater in the UME group than in the non-UME group (443.66 ± 142.29 vs. 265.94 ± 19.68 μm, *P* < 0.001). The configuration of UME categorized by infectious and noninfectious uveitis is summarized in Table 2. DiffME alone was the most frequent finding (36.5%), followed by the combined form of SRD, and CME/DiffME (32.6%), and CME (30.9%). No significant differences in the configuration of UME were found between infectious and noninfectious uveitis (*P* = 0.966). Regarding the diagnosis, idiopathic uveitis was the most common cause of all subtypes of UME. The most frequent identifiable causes of CME, DiffME, and SRD were tuberculous uveitis, cytomegalovirus retinitis, and Vogt-Koyanagi-Harada disease, respectively.

**Table 2:** Characteristics of uveitic macular edema categorized by infectious and noninfectious uveitis

Parameters	Total	Infection	Noninfection
	(*N* = 181)	(*n* = 85)	(*n* = 96)
Cystoid macular edema alone	56 (30.9)	27 (31.8)	29 (30.2)
Diffuse macular edema alone	66 (36.5)	31 (36.5)	35 (36.5)
Serous retinal detachment^a^	59 (32.6)	27 (31.8)	32 (33.3)

^a^ Presence of serous retinal detachment combined with cystoid macular edema or diffuse macular edema

Predictive factors associated with UME are shown in Table 3. In multivariate analysis, increasing age at the onset of uveitis was significantly associated with UME (adjusted odds ratio [aOR] 1.01, 95% confidence interval [CI] 1.00-1.03, *P* = 0.036). PU and panuveitis were significantly correlated with UME compared to intermediate uveitis (aOR 2.09, 95% CI 1.14–3.86, *P* = 0.018). Uveitis of infectious etiology was also significantly associated with UME compared to noninfectious uveitis (aOR 2.13, 95% CI 1.34–3.37, *P* = 0.001).

**Table 3:** Univariate and multivariate analyses of predictive factors associated with uveitic macular edema

Parameters	Univariate analysis			Multivariate analysis		
	OR	95% CI	Pvalue	aOR	95% CI	*P* value^a^
Age of uveitis onset	1.01	1.00-1.03	0.064	1.01	1.00-1.03	0.036*
Female gender	0.73	0.48–1.12	0.146			
Bilateral uveitis	0.74	0.48–1.13	0.160			
Chronic uveitis	0.69	0.43–1.12	0.132			
Posterior and panuveitis^b^	2.43	1.35–4.38	0.003*	2.09	1.14–3.86	0.018*
Granulomatous inflammation	0.97	0.64–1.47	0.871			
Infectious uveitis^c^	2.35	1.5–3.67	< 0.001*	2.13	1.34–3.37	0.001*

aOR adjusted odds ratio; CI confidence interval; OR odds ratio; UME uveitic macular edema

^a^*P*-value of Wald test

^b^ Compared to intermediate uveitis

^c^ Compared to noninfectious etiology

## Discussion

This retrospective analysis of UME in 349 patients with intermediate, posterior and panuveitis demonstrated that half of uveitis patients experienced UME. The majority of patients with UME presented with chronic, bilateral uveitis. UME was associated with older age at the onset of uveitis, posterior and panuveitis, and infectious etiology.

This study showed a comparable prevalence of UME when compared to other studies that reported rates ranging from 33 to 48% [3, 5, 13]. In the present study, UME accounted for 51.9% of uveitis patients, which was derived from 27.5% of noninfectious causes and 24.4% of infectious causes. The slightly higher rate of UME in this study was explained by the difference in uveitis diagnosis, with a relatively higher proportion of infectious uveitis with UME. UME has been shown to be an important factor associated with visual loss in up to 42% of patients with uveitis [2, 3].

Our analysis revealed that UME occurred more commonly in patients with PU (57%) and panuveitis (54%) than in those with IU (34%). This finding is contrary to that of several studies that have reported that IU (35–60%) and panuveitis (18–66%) are frequent forms associated with UME [3, 4, 6, 11]. Regarding predictive factors associated with UME, PU and panuveitis were significantly associated with UME compared with IU. It is possible that infectious uveitis, which was a relatively common etiology of UME in this study and tended to present with PU, contributed to the different results.

The observation that older age at disease onset independently predicted UME has been noted in previous studies, and our data showed this as well [3, 5, 13]. A study of 97 patients with uveitis observed a strong association between advancing age and UME (3.8-fold higher in patients > 50 years and 4.5-fold higher risk in patients > 70 years) [13]. The results of the present study showed that the likelihood of developing UME increased by 1% for every 1-year increase in age. Increasing age might impair physiological clearance of retinal edema or be associated with other ocular comorbidities not otherwise adjusted for in the analysis [5].

The finding that infectious uveitis independently predicted UME in our population is consistent with that of previous studies [14, 15]. The majority of our tuberculous uveitis patients presented with occlusive retinal peri-phlebitis with secondary branch vein occlusion, a complication that itself is associated with macular edema [14]. A study from Tunisia found a high 42% rate of UME among their presumed tuberculous uveitis patients [15]. Lardenoye et al. reported that one of the most common inflammatory eye diseases related to UME was acute retinal necrosis, although ocular tuberculosis was not among their patients’ diagnoses [3]. We suspect that the high rate of UME among our patients with infectious uveitis is related to the more severe degree of inflammation and our cautious approach to corticosteroid use in this group. This hesitation likely results in a longer duration of inflammation, leading to UME as a consequence.

The retrospective nature of this study with inevitable patient selection bias and data inconsistencies, along with wide variability in follow-up durations, naturally limits the generalizability of our findings. The strengths of our study include the large sample size, which was sufficient to detect significant differences. Nevertheless, if our results do, in fact, represent a pathobiologic reality such that age, posterior/panuveitis, and infectious etiology predict the development of UME, these findings could meaningfully affect the management of patients with uveitis by inclining colleagues to head off this complication with earlier or more aggressive interventions in at-risk patients. We believe that our findings point to the likely utility of exploring these putative risk factors with more rigorously designed prospective interventional studies.

## Limitations

Regrettably, we did not gather precise data regarding the average duration of macular edema following the onset of uveitis, nor did we document comprehensive details on the anti-inflammatory treatments administered to our patients. Consequently, we are currently unable to furnish this information. Acknowledging that suboptimal anti-inflammatory treatment and prolonged duration of intraocular inflammation are known risk factors for macular edema, it is essential to bear this information in mind. Further investigation, focusing on the detailed development and treatment of uveitic macular edema, would be advantageous. Nevertheless, anterior uveitis was not included in the present study because we did not regularly perform SD-OCT on patients with anterior uveitis.

## Conclusions

The prevalence of UME in intermediate, posterior and panuveitis was relatively high in our setting. Increasing age at the onset of uveitis, posterior and panuveitis, and infectious uveitis were the predictive factors associated with UME.

## Data Availability

The full trial protocol, datasets used and/or analyzed during the current study are available from the corresponding author on reasonable request.

## Abbreviations

ARN: acute retinal necrosis
CME: cystoid macular edema
CMVR: cytomegalovirus retinitis
DiffME: diffuse macular edema (DiffME)
HLA: human leukocyte antigen
IU: intermediate uveitis
PORN: progressive outer retinal necrosis
PU: posterior uveitis
SD-OCT: spectral domain optical coherence tomography
SNSA: seronegative spondyloarthropathy
SRD: serous retinal detachment
UME: uveitic macular edema
